# Absence of a Faster-X Effect in Beetles (*Tribolium*, Coleoptera)

**DOI:** 10.1534/g3.120.401074

**Published:** 2020-01-27

**Authors:** Carrie A. Whittle, Arpita Kulkarni, Cassandra G. Extavour

**Affiliations:** * Department of Organismic and Evolutionary Biology, and; † Department of Molecular and Cellular Biology, Harvard University, 16 Divinity Avenue, Cambridge MA 02138

**Keywords:** *Tribolium castaneum*, faster-X, sex-biased expression, dosage compensation, dN/dS

## Abstract

The faster-X effect, namely the rapid evolution of protein-coding genes on the X chromosome, has been widely reported in metazoans. However, the prevalence of this phenomenon across diverse systems and its potential causes remain largely unresolved. Analysis of sex-biased genes may elucidate its possible mechanisms: for example, in systems with X/Y males a more pronounced faster-X effect in male-biased genes than in female-biased or unbiased genes may suggest fixation of recessive beneficial mutations rather than genetic drift. Further, theory predicts that the faster-X effect should be promoted by X chromosome dosage compensation. Here, we asked whether we could detect a faster-X effect in genes of the beetle *Tribolium castaneum* (and *T. freemani* orthologs), which has X/Y sex-determination and heterogametic males. Our comparison of protein sequence divergence (dN/dS) on the X chromosome *vs.* autosomes indicated a rarely observed absence of a faster-X effect in this organism. Further, analyses of sex-biased gene expression revealed that the X chromosome was particularly highly enriched for ovary-biased genes, which evolved slowly. In addition, an evaluation of male X chromosome dosage compensation in the gonads and in non-gonadal somatic tissues indicated a striking lack of compensation in the testis. This under-expression in testis may limit fixation of recessive beneficial X-linked mutations in genes transcribed in these male sex organs. Taken together, these beetles provide an example of the absence of a faster-X effect on protein evolution in a metazoan, that may result from two plausible factors, strong constraint on abundant X-linked ovary-biased genes and a lack of gonadal dosage compensation.

The “faster-X” effect, that is, the rapid evolution of protein-coding genes on the X chromosome, has been widely reported in a range of metazoan systems with sex chromosomes (Charlesworth *et al.* 1987; Meisel and Connallon 2013). Higher rates of protein divergence of genes on the hemizygous X chromosome (faster-X, or faster-Z in W/Z systems) than on autosomes has been observed in organisms including primates (Lu and Wu 2005; Stevenson *et al.* 2007), humans (Lu and Wu 2005), rodents (Baines and Harr 2007; Kousathanas *et al.* 2014), birds (Mank *et al.* 2007b; Mank *et al.* 2010a), moths (Sackton *et al.* 2014), aphids (Jaquiéry *et al.* 2018), and very recently in spiders (Bechsgaard *et al.* 2019). In other organisms, however, a faster-X effect is more ambiguous. For example, signals of this effect have sometimes, but not always, been observed in studies of fruit flies (Mank *et al.* 2010b; Meisel and Connallon 2013; Avila *et al.* 2014; Charlesworth *et al.* 2018), and variable results on the presence or strength of the faster-X effect have been reported in butterflies (Rousselle *et al.* 2016; Pinharanda *et al.* 2019).

With regards to the mechanisms that might account for the faster-X effect, it has been proposed that X-linked genes may evolve faster in protein sequence than those on autosomes due to efficient fixation of recessive beneficial mutations in the hemizygous state, a notion that has found empirical support in some animal taxa (Charlesworth *et al.* 1987; Lu and Wu 2005; Baines and Harr 2007; Baines *et al.* 2008; Meisel and Connallon 2013; Campos *et al.* 2018; Charlesworth *et al.* 2018). An alternative mechanism is that the effect results largely from fixation of recessive mildly deleterious mutations via genetic drift. Studies in birds for example support this mechanism, which has been suggested to be facilitated by low effective population size (Charlesworth *et al.* 1987; Mank *et al.* 2010a; Mank *et al.* 2010b; Parsch and Ellegren 2013).

The study of sex-biased gene expression, that is, those genes preferentially upregulated in one sex, has helped to decipher the forces shaping the molecular evolutionary rates on the X chromosome *vs.* autosomes (Ranz *et al.* 2003; Kirkpatrick and Hall 2004; Zhang *et al.* 2004; Assis *et al.* 2012; Campos *et al.* 2018), and thus the faster-X effect (Baines *et al.* 2008; Mank *et al.* 2010a; Parsch and Ellegren 2013; Avila *et al.* 2014; Sackton *et al.* 2014; Rousselle *et al.* 2016; Pinharanda *et al.* 2019). For instance, in male heterogametic organisms, under a model wherein the faster-X effect is caused by rapid fixation of recessive beneficial mutations in the hemizygous state (wherein phenotypes are not masked by non-mutant alleles), this effect is predicted to be strongest in male-biased genes, and relatively lower in female-biased and unbiased genes (Baines *et al.* 2008; Mank *et al.* 2010a; Parsch and Ellegren 2013). Empirical support for this model comes from a study of *Drosophila*, in which assessment of protein divergence (dN/dS) of genes showed a faster-X effect for all three classes of sex biased genes (male-biased, female-biased and unbiased), an effect largest in magnitude for male-biased genes (Baines *et al.* 2008; Parsch and Ellegren 2013). In chickens, which have W/Z sex chromosomes and female heterogamety, elevated dN/dS has been reported across studied genes on the Z-chromosome, consistent with the faster-X (or faster-Z in this case) effect (Mank *et al.* 2007a). However, the prediction of higher dN/dS for female-biased genes on the Z-chromosome was not met, and thus faster-Z in these birds was linked to fixation of neutral or slightly deleterious mutations via genetic drift (Mank *et al.* 2010a; Parsch and Ellegren 2013). Recently, similar results were reported for the W/Z chromosomes of *Heliconius* butterflies (Pinharanda *et al.* 2019). In this regard, sex-biased gene expression may help ascertain the mechanisms underlying a faster-X effect.

The faster-X effect may be expected to be most strongly observed in organisms with complete dosage compensation, wherein expression levels of X-linked genes are upregulated in the heterogametic sex, such that the X to autosome ratio (X:A) is one or close to one (Charlesworth *et al.* 1987; Mank *et al.* 2010b; Kayserili *et al.* 2012; Meisel and Connallon 2013). Under this hypothesis, in organisms with incomplete X chromosome dosage compensation, such that X:A <1, X-linked recessive beneficial mutations would have relatively low expression levels, and thus putatively weak phenotypic effects (or selection coefficients (Charlesworth *et al.* 1987)), in the hemizygous sex. This could make beneficial mutations exposed on the single male X chromosome unlikely to be fixed any more frequently than if they were autosomal, possibly minimizing a faster-X effect (Charlesworth *et al.* 1987; Mank *et al.* 2010a). The notion that dosage compensation may impact the rates of X- (or Z-) linked gene evolution is consistent with the observation that certain butterflies display incomplete dosage compensation (Walters *et al.* 2015), and they also lack the elevated faster-Z effect expected in female-biased genes (Pinharanda *et al.* 2019), see also results for birds (Mank *et al.* 2010a). At present however, the relationship between faster-X effect and dosage compensation remains only rarely empirically evaluated (Mank *et al.* 2010b).

One understudied and significant facet of male dosage compensation in an organism is that this phenomenon may vary among tissue types. For instance, studies in *Drosophila* have shown that complete dosage compensation of X-linked genes is observed in male somatic tissues, but not in the male germ cells or testis (Vibranovski *et al.* 2009; Meiklejohn and Presgraves 2012; Argyridou and Parsch 2018). In the context of such findings in *Drosophila*, it may be speculated that the purported weak faster-X effect observed in that taxon (Mank *et al.* 2010b; Meisel and Connallon 2013; Avila *et al.* 2014; Charlesworth *et al.* 2018) may be connected its poor testis dosage compensation (Charlesworth *et al.* 1987). In this regard, studies of the faster-X effect in animals should thus consider dosage compensation in specific tissues, such as gonadal and non-gonadal dosage compensation.

A model insect genus that offers new opportunities to study the faster-X effect is the beetle system *Tribolium* (Coleoptera). Coleoptera is the largest insect order, with recent estimates of over 1.5 million species, and comprising approximately 40% of all arthropod species (Stork *et al.* 2015). The rust red flour beetle *T. castaneum* is a well-established model system for genetics and for the evolution of developmental mechanisms (Brown *et al.* 1994; Savard *et al.* 2006; Denell 2008; Brown *et al.* 2009; Choe *et al.* 2017), and has extensive genomic resources available for research (Wang *et al.* 2007; Tribolium Genome Sequencing Consortium *et al.* 2008; Williford and Demuth 2012). In addition, its less well-studied sister species *T. freemani*, from which it diverged approximately 12-47 Mya, comprises a suitable system for comparative genomic study (Angelini and Jockusch 2008). To date, however, to our knowledge the primary genome-wide sex-biased expression research in *Tribolium* that includes X chromosome analyses consists of a foundational study based on whole male *vs.* whole female contrasts and microarray data in *T. castaneum* (Prince *et al.* 2010). That assessment made several significant findings, including that female-biased genes were highly overrepresented on the X chromosome (Prince *et al.* 2010), and was thought to comprise an imperfect response to male dosage compensation (Prince *et al.* 2010). In addition, the study authors reported that X-linked genes with male-biased expression were comparatively uncommon, a trend also observed to some extent in other organisms such as *Drosophila* (Prince *et al.* 2010). Other transcriptome and genomic studies in *T. castaneum* include assessments of differential expression among somatic, germ line, and embryonic tissues (Khan *et al.* 2019) and its codon and amino acid usage (Williford and Demuth 2012; Whittle *et al.* 2019). None of these studies however assessed evidence for or against the faster-X effect in *Tribolium*. Moreover, there is a lack of between-species analyses of protein sequence divergence (dN/dS) and its potential relationship to sex-biased gene expression and dosage compensation.

Here, we describe a rigorous assessment of the faster-X effect in *T. castaneum*, including evaluation of its relationship to sex-biased gene expression and dosage compensation, using newly generated RNA-seq data from gonads and gonadectomized (GT-) males and females. Our assessment of dN/dS in 7,751 *T. castaneum* genes with high confidence orthologs in its sister taxon *T. freemani* reveals the absence of a faster-X effect in this taxon. Instead, we find a tendency for a slower rate of protein sequence evolution of X-linked as compared to autosomal genes. Further, we show that the faster-X effect (using dN/dS) is not found for male-biased, female-biased or unbiased genes from the gonads or from non-gonadal somatic tissues. We demonstrate that the slow-X effect in this taxon is largely linked to ovary-biased genes located on the X chromosome, which are more common, and have evolved more slowly, than those on autosomes. In addition, we report that while somatic tissues of males (GT-males) exhibit nearly complete dosage compensation, a striking lack of X chromosome dosage compensation is observed in the testis, which may limit the fixation of recessive beneficial mutations (Charlesworth *et al.* 1987), and possibly contribute toward the lack of a faster-X effect in this taxon.

## Materials and Methods

### CDS per species and defining orthologs

The previously annotated protein-coding genes in our main target taxon *T. castaneum* were downloaded for study (v. 5.2, Ensembl Metazoa (http://metazoa.ensembl.org (Wang *et al.* 2007; Tribolium Genome Sequencing Consortium *et al.* 2008)). For the genome of *T. freemani*, which we used as a reference to determine dN/dS, CDS have not previously been annotated and thus were extracted from available scaffolds. The scaffold assembly was downloaded from BeetleBase (version 4, http://www.Beetlebase.org, (Wang *et al.* 2007)). Details on identification of CDS for *T. freemani* are provided in Supplemental Text File S1.

In the final gene list for *T. castaneum* and for *T. freemani*, only those CDS (longest CDS per gene used for study) having a start codon, not having unknown or ambiguous nucleotides or internal stop codons, and ≥33 amino acids were retained for study. The total number of CDS after filtering was 16,434 for *T. castaneum* (average GC content of protein coding genes was 46.1% (±5X10^−4^ standard error)) that is marginally more than the 16,404 gene models first defined for this species (Tribolium Genome Sequencing Consortium *et al.* 2008). A total of 12,628 CDS were obtained for the sister species *T. freemani*.

### Gene expression and identification of sex-biased genes

#### Biological samples and RNA-seq:

*T. castaneum* and *T. freemani* specimens were provided by the Brown lab at Kansas State University (strain IDs; https://www.k-state.edu/biology/people/tenure/brown/). Samples were grown under standard laboratory conditions until adulthood as previously described (Brown *et al.* 2009). Technical details on tissue collection, PCR, and RNA-seq are provided in Supplemental Text File S1. RNA-seq samples are described in Table S1.

For males, the isolated reproductive tissues included the testes, accessory glands (mesadenia, ectadenia), and directly attached tissues (vesicular seminalis, vas deferens and ejaculatory duct) while for females, gonad samples included the ovaries and their linked tissues (spermathecal gland, common oviduct, spermathecae, and vagina). For simplicity, we refer to the male and female reproductive organs and tissues collectively as “testis” and “ovary” or the sex-neutral “gonads” herein, with the understanding that they include the abovementioned reproductive tissues directly linked to the respective gonads. All remaining non-gonadal tissues of the adult body are referred to as the gonadectomized (GT-) soma, or GT-males and GT-females. For the sister species *T. freemani*, four RNA-seq samples, one per tissue-type, testes, ovaries, GT-males and GT-females, were obtained and used for refining the CDS list for this species (see Supplemental Text File S1).

The RNA-seq reads (76bp) per sample type (Table S1) were trimmed of adapters and poor-quality bases using the program BBduk available from the Joint Genome Institute (https://jgi.doe.gov/data-and-tools/bbtools/). Gene expression level per gene was determined by mapping each RNA-seq dataset per tissue to the full CDS list for each species using Geneious Read Mapper, a program that provides similar read match performance as other common read-mappers such as Bowtie (Langdon 2015) or BBmap (https://jgi.doe.gov/, data not shown, also (Whittle and Extavour 2019)). Read counts per CDS were converted to FPKM for each gene. The replicates per tissue type had Spearman’s R > 0.91 for FPKM across all genes (*P* < 2X10^−7^). Expression was compared between the testes and ovaries, and between GT-males and GT-females by using Deseq2 to obtain P-values (Love *et al.* 2014) and the average FPKM of the replicates per tissue type (Table S1). Any gene having at least a twofold difference in average expression (Mank *et al.* 2010a; Assis *et al.* 2012; Whittle and Extavour 2017) and having a statistically significant P-value in Deseq2 (*P* < 0.05) as well as a FPKM of at least one in one tissue type was identified as sex-biased. All other genes were defined as unbiased.

### Ortholog identification and sequence divergence

Using the *T. castaneum* CDS list we identified 7,751 high confidence orthologs in its sister species *T. freemani* for our study of protein sequence evolution (dN/dS; note that while the core analyses of dN/dS involve these 7,551 genes, the expression results for all 16,434 *T. castaneum* genes are described throughout when appropriate). The use of closely related sister species is a common approach to study the protein sequence divergence of sex-biased genes in metazoan models (*cf*. (Mank *et al.* 2007b; Baines *et al.* 2008; Meisel 2011; Grath and Parsch 2012; Perry *et al.* 2015; Jaquiéry *et al.* 2018)). Values of dN/dS <1, =1, and >1 suggest that purifying, neutral and positive selection respectively are likely to predominantly shape the evolution of protein coding genes (Yang 2007). However, even when dN/dS <1 (as is typical in gene-wide analysis), relatively elevated values suggest reduced constraint, which could be due to relaxed selection and/or adaptive evolution.

The 7,751 orthologs between *T. castaneum* and *T. freemani* for dN/dS analysis were identified using reciprocal BLASTX of the full CDS list per species in the program BLAST+ v2.7.1 (https://blast.ncbi.nlm.nih.gov). Only genes having the same best match in both forward and reverse contrasts between species and an e-value <10^−6^ were defined as orthologs. In the rare cases when two CDS had the same e-value, the one with the highest bit score was taken as the best match. For additional stringency in the study of dN/dS, only those genes that were reciprocal BLASTX best matches and where dN and dS values of alignments (≥33 amino acids) were each <1.5 (Castillo-Davis *et al.* 2004; Treangen and Rocha 2011), were identified as orthologs between *T. castaneum* and *T. freemani* (note also that across all 7,751 genes, the median dN and dS values were 0.026 and 0.291 respectively, and thus markedly below saturation). Thus, the alignments and dN/dS measures herein are conservative.

Orthologous gene sequences in *T. freemani* and *T. castaneum* were aligned by codons using MUSCLE set to default parameters in the program Mega-CC v7 (Kumar *et al.* 2012). Alignments were then filtered to remove gaps. It has been suggested that removal of highly divergent segments from alignments, while causing loss of some sequence regions, improves measurements of protein sequence divergence; thus, highly divergent segments were excluded using the program Gblocks v. 0.91b set at default parameters (Castresana 2000; Talavera and Castresana 2007). Each gene alignment was then run in yn00 of PAML, which accounts for codon usage biases (Yang 2007), to measure dN, dS, and dN/dS (Yang 2007).

### X chromosomes vs. autosomes

Chromosomal locations of genes are available in the annotation for *T. castaneum* (http://metazoa.ensembl.org, also available at BeetleBase (Wang *et al.* 2007; Tribolium Genome Sequencing Consortium *et al.* 2008)). The Y-chromosome of *T. castaneum* is small (<5MB), highly degenerate, contains few if any protein- coding genes, and is not included in the genetic linkage map; accordingly it was not studied (Tribolium Genome Sequencing Consortium *et al.* 2008; Brown *et al.* 2009; Prince *et al.* 2010; Shukla and Palli 2014).

### Gene ontology

Gene ontology (GO) was assessed using DAVID software (Huang Da *et al.* 2009). For this, we identified orthologs to *T. castaneum* in the reference insect model *D. melanogaster* (CDS v6.24 available from www.flybase.org) (Gramates *et al.* 2017) using BLASTX (https://blast.ncbi.nlm.nih.gov) to identify the best match (lowest e-value with cut off of e < 10^−6^). *D. melanogaster* gene identifiers, which are accepted as input into DAVID, were used to obtain GO functions for *T. castaneum* genes. Single direction BLASTX with *T. castaenum* CDS as the query to the *D. melanogaster* database was used for this assessment (unlike for the reciprocal BLASTX between *Tribolium* species), as we considered reciprocal BLASTX to be overly stringent between these divergent insects (which are from different orders) for the purpose of GO functional analysis.

### Data availability

The CDS v. 5.2 for *T. castaneum* are available at Ensembl Metazoa (http://metazoa.ensembl.org). Scaffolds for *T. freemani* are available at BeetleBase (Wang *et al.* 2007; Tribolium Genome Sequencing Consortium *et al.* 2008). RNA-seq data and SRA Biosample identifiers for all 12 samples from *T. castaneum* and *T. freemani* described in Table S1 are available at the SRA database under Bioproject accession number PRJNA564136. All cited Supplemental Materials (tables, figures, text files) are available at Figshare: https://doi.org/10.25387/g3.11699796.

## Results

**Figure 1:**
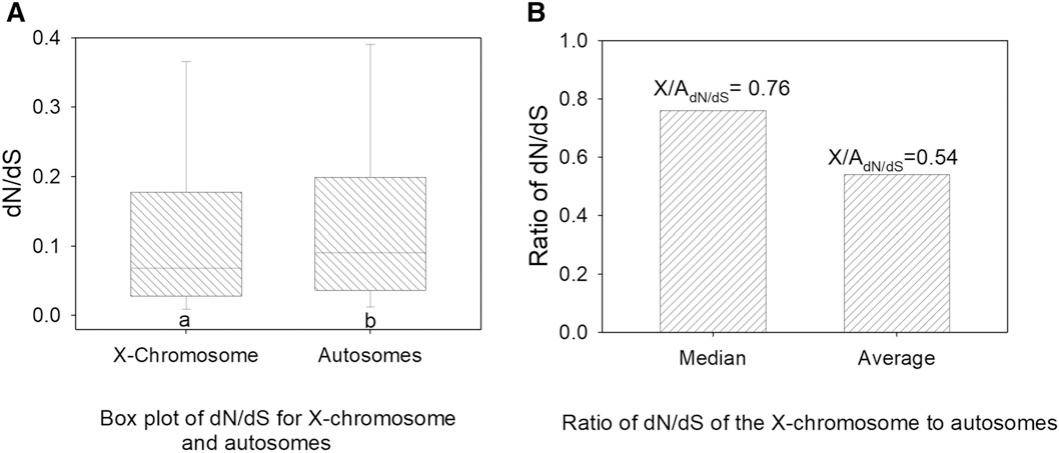
The dN/dS of genes located on the X chromosome *vs.* autosomes. A) Box plots of dN/dS showing the median, upper and lower quartiles, and 95/5^th^ percentiles; B) the ratio of dN/dS for the X chromosome *vs.* the autosomes using the median and mean values per group. Different letters under bars in panel A indicate a statistically significant difference using Mann-Whitney U (MWU)-tests.

We first assessed whether the beetle system exhibited a faster-X effect, in terms of higher dN/dS on the X chromosome than autosomes (Lu and Wu 2005; Mank *et al.* 2007a; Baines *et al.* 2008; Parsch and Ellegren 2013; Pinharanda *et al.* 2019). Box plots of dN/dS for genes located on the X chromosome and autosomes using the 7,751 genes with inter-species orthologs are shown in Figure 1A. The results showed no tendency for higher dN/dS in genes on the X chromosome. In fact, the opposite was observed: dN/dS was statistically significantly lower for X-linked genes than for autosomal genes in this taxon (MWU-test *P* = 0.002). From a total of 432 studied X-linked genes and 7,319 autosomal genes distributed across nine autosomes, the median dN/dS values were 0.0686 (95% confidence interval of 0.0624-0.0726, Table 1) and 0.0908 (95% confidence interval of 0.8009-0.1024) respectively (Figure 1A), yielding a ratio of median dN/dS values for the X chromosome to autosomes across all genes (X/A_dN/dS (all genes)_) of 0.76 (Figure 1B). Further, the mean dN/dS on the X chromosome was about half (ratio of 0.54) that observed on autosomes (Figure 1B), and thus was also considerably below 1. Collectively, these results indicate the absence of a faster-X effect on interspecies dN/dS in this taxon, differing from that observed in most other comparable metazoan studies to date.

**Table 1:** The median dN/dS values for genes located on the X chromosome and pooled autosomes, and the 95% confidence intervals for each median. Values are shown for all genes in *T. castaneum* under study (with identifiable orthologs in *T. freemani*, N = 7,751), sex-biased gonadal genes, sex-biased nongonadal genes, and unbiased genes (Figure 2CD). Confidence intervals were determined using bootstrapping with 1,000 replicates. N values per category are shown in Table S2

Chromosome(s)	All Genes	Gonads			GT-soma		
		Ovary-biased	Testis-biased	Gonadal-unbiased	GT-female biased	GT-Male biased	GT-unbiased
X chromosome median	0.0686	0.0603	0.0890	0.0946	0.0738	0.0486	0.0681
95% CI	0.0624-0.0726	0.0500-0.0667	0.0341-0.1937	0.0738-0.1163	0.0556-0.1248	0.0185-0.1175	0.0624-0.0799
Pooled autosomes median	0.0908	0.0850	0.1204	0.0874	0.1421	0.1069	0.0880
95% CI	0.8009-0.1024	0.0804-0.0909	0.1139-01304	0.0843-0.0874	0.1212-0.1604	0.0971-0.1165	0.0852-0.0908

### Assessment of sex-biased genes on the X chromosome *vs.* autosomes

Having found no evidence of a faster-X effect using dN/dS for this beetle taxon, we next asked if sex-differences in gene expression could help suggest mechanisms that might explain this pattern (Figure 1) (Baines *et al.* 2008; Mank *et al.* 2010a; Parsch and Ellegren 2013; Avila *et al.* 2014; Rousselle *et al.* 2016; Pinharanda *et al.* 2019). For the 16,434 genes in *T. castaneum*, we found that 25.8% had gonad-biased expression (N = 4,232), and 9.6% of genes (N = 1,573) had biased expression in the GT-soma (Figure S1). The N values of sex-biased genes for those genes with interspecies orthologs (N = 7,751) are shown in Figure S2 (N = 2,341 (30.2%) and 836 (10.7%) for gonads and GT-soma respectively). Using the sex-biased gene sets, and the sexually unbiased genes, we further assessed dN/dS of X-linked and autosomal genes.

**Figure 2:**
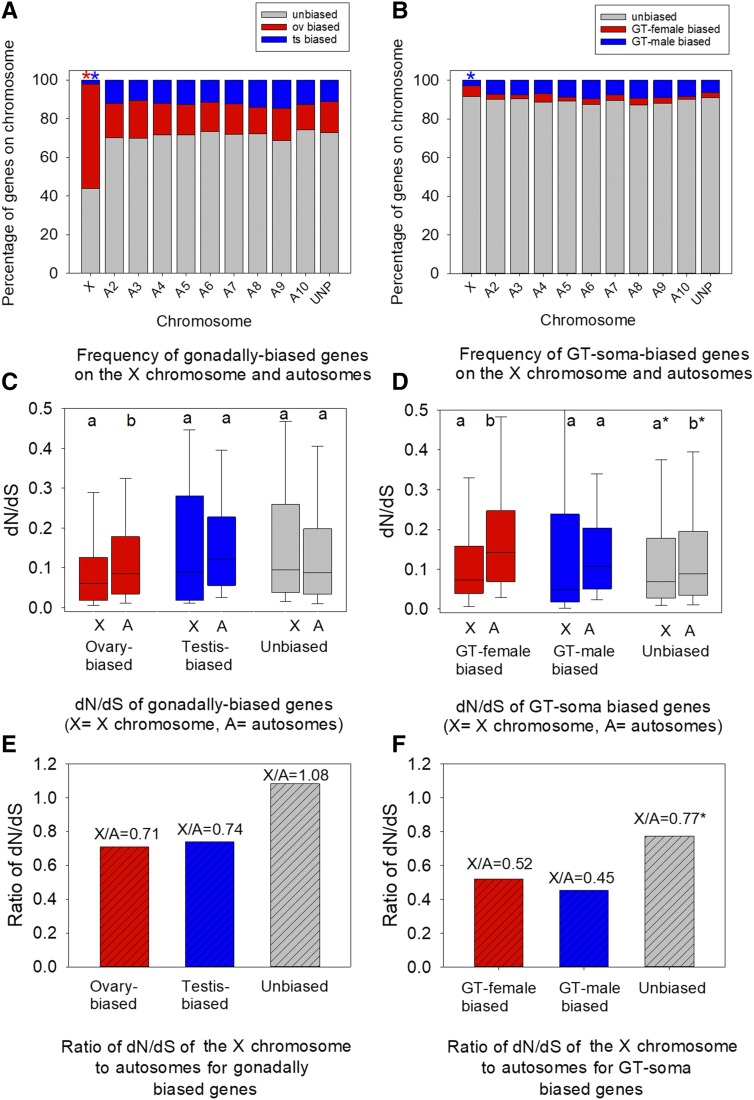
Assessment of the faster-X effect with respect to sex-biased genes in *T. castaneum*. A) The frequency of gonadally sex-biased genes on the X chromosome and nine autosomes for the 7,751 genes under study; B) the frequency for GT-soma sex-biased genes; C) box plots of the dN/dS of ovary-biased, testis-biased and unbiased genes on the X chromosome and autosomes; D) the dN/dS of GT-male biased, GT-female biased, and GT-unbiased genes on the X chromosome and autosomes; E) the ratio of the median dN/dS of the X chromosome to the autosomes (X/A_dN/dS_) for all three categories of sex-biased expression for the gonads; and F) for the GT-soma. In A, the red and blue asterisks indicate more ovary-biased and fewer testis-biased (or GT-male biased in B) genes were located on the X chromosomes than on pooled autosomes (Chi^2^-P with Yates’ correction *P* < 0.05 for each contrast). Different lowercase letters on top of each pair of bars in C and D indicate MWU-test *P* < 0.05. In C-F, unmapped genes were included with autosomal genes and their inclusion in or exclusion from the analysis yielded similar results. *Note that differences in X-linked and autosomal unbiased genes in panels D and F are explained by ovary-biased genes as outlined in the main text. ov=ovary, ts=testis.

The proportion of genes on the X chromosome and on each of the nine autosomes that had sex-biased or unbiased expression is shown in Figure 2A, which includes all genes for which we had calculated dN/dS values (N = 7,751) (see Figure S3 for all 16,434 annotated *T. castaneum* genes, which yielded similar patterns according to sex-biased expression status). We found that a disproportionately large fraction of genes on the X chromosome were ovary-biased: 53.9% of the X-linked genes under study were ovary-biased (N = 233 of the 432 X-linked genes for which we assessed dN/dS) (Figure 2A), while only 16.3% of autosomal genes showed ovary-biased expression (N = 1,192 of 7,319 genes pooled across autosomes, Chi^2^ with Yates’ correction *P* < 0.0001). In contrast, relatively few testis-biased, GT-male biased or GT-female biased genes were located on the X chromosome (each of these gene expression categories constituted ≤5.5% of the X-linked genes under study in Figure 2AB). These results for the 7,751 genes with orthologs between *T. castaneum* and *T. freemani* (Figure 2AB) parallels that found for all *T. castaneum* genes in the genome (Figure S3AB). The patterns in expression concur with a prior report on *T. castaneum*, which used transcriptome data from whole males and females to show a concentration of female-biased genes and rarity of male-biased genes on the X chromosome (Prince *et al.* 2010). However, our present expression findings explicitly show that ovary-biased genes (Figure 2A) are highly concentrated on the X chromosome, and that X-linked testis-biased genes, GT-male-biased, and GT-female-biased genes are each relatively uncommon on the X chromosome.

#### The absence of a faster-X effect and the presence of slowly evolving X-linked ovary-biased genes:

Having identified that ovary-biased genes were highly overrepresented on the X chromosome (Figure 2A), we asked if this might contribute to the observed slower-X effect. We compared dN/dS values for these ovary-biased genes on X chromosomes to those values for autosomal ovary-biased genes (Figure 2CE; N values in Table S2, Figure S2). We found that the dN/dS values (median = 0.0603) of X-linked ovary-biased genes were statistically significantly lower than dN/dS values for autosomal ovary-biased genes (median = 0.0850; MWU-test *P* < 0.001, Figure 2C). The 95% confidence intervals of the medians were non-overlapping, with values of 0.0500-0.0667 for the former X-linked ovary-biased gene set, and 0.0804-0.0909 for the latter autosomal group (Table 1). Thus, the faster-X effect in terms of dN/dS is not observed for ovary-biased genes. Further, the ratio of the median dN/dS values when calculated using only the subset of X-linked ovary-biased genes *vs.* those on autosomes, X/A_dN/dS (ovary-biased)_, was 0.71 (Figure 2E), also suggesting slower evolution of ovary-biased genes on the X chromosome than autosomes. Moreover, ovary-biased genes on the X chromosome had lower dN/dS than gonadally unbiased genes on the X chromosome and on autosomes (MWU-tests *P* < 0.001), and than testis-biased genes on the autosomes (MWU-test *P* < 0.001; note there were too few X-linked testis-biased genes for reliable statistical testing of that contrast, Table 1, Figure 2C, Table S2). Together, given the marked abundance of ovary-biased genes on the X chromosome (Figure 2A), and their low dN/dS values (Figure 2C, Table 1), it is evident that these genes contribute toward the lack of a faster-X effect in this organism (Figure 1).

For the genes with GT-soma-biased expression, there were only 24 genes with GT-female biased expression on the X chromosome (as compared to 233 with ovary-biased expression on the X chromosome, Table S2). Nonetheless, as we had observed for ovary-biased genes, this small number of GT-female biased genes also had statistically significantly lower dN/dS values than the GT-female biased genes on autosomes (MWU-test *P* = 0.031, Figure 2D; 95% confidence intervals in Table 1), and the X/A_dN/dS (GT-female)_ value when calculated for this subset of genes was also low, at 0.52 (Figure 2F). Thus, it appears that there has also been strong purifying selection on X-linked GT-female biased genes in this taxon. Upon close examination however, and as shown in Table S2, 17 of the 24 (70.8%) X-linked GT-female biased genes also had ovary-biased expression, suggesting that the observed effect could be due to purifying selection arising from ovarian expression rather than somatic expression. Nonetheless, the seven genes with GT-female biased but not ovary-biased expression yielded a X/A_dN/dS (GT-female)_ (median) ratio of 0.32, suggesting that X-linked GT-female-biased genes are under stronger constraint than those on autosomes, regardless of their ovary-biased expression status. Thus, we find no evidence of a faster-X effect for any female-biased genes, including those with gonadal or somatic expression.

We next assessed whether the faster-X effect was observable for male-biased genes (testis- or GT-male-biased), which would be expected to exhibit a pronounced faster-X effect under a hypothesis of rapid fixation of beneficial recessive mutations in the heterogametic sex (Mank *et al.* 2010a; Parsch and Ellegren 2013). We found that very few testis-biased genes or GT-male-biased genes were located on the X chromosome (N = 9 and N = 12 for testis-biased and GT-male-biased X-linked genes with interspecies orthologs), and that neither group of male-biased genes showed a pattern consistent with a faster-X effect. The median dN/dS value was lower for these genes on the X chromosome than on autosomes for both categories of genes (Figure 2CD). The X/A_dN/dS (testis-biased)_ ratio was 0.74 for testis-biased genes, and the X/A_dN/dS (GT-male biased)_ ratio was 0.45 for GT-male biased genes (Figure 2EF), markedly below 1 in both cases. No overlap was observed between the testis-biased and GT-male biased gene sets (Table S2), and thus the low dN/dS effects were independently observed in each group. For stringency, we examined and noted that three of the GT-male-biased genes were also ovary-biased, but exclusion of those genes from the analysis still yielded an X/A_dN/dS (GT-male biased)_ ratio of 0.59, and thus the low dN/dS effect is directly linked to the GT-male-biased expression. In sum, while the small number of X-linked testis-biased and GT-male-biased genes precludes rigorous statistical testing of those genes, such that these particular contrasts remain anecdotal (in addition to these samples having wide confidence intervals (Table 1)), we note that the patterns observed for these genes are inconsistent with a faster-X effect in male-biased genes, whether gonad- or soma-biased.

We next asked whether there was evidence for the faster-X effect in the gonadally unbiased genes. Given that such genes were common on all chromosomes (Figure 2A, Table S2), which provides the potential for high statistical power, and that they by definition exclude the slow evolving X-linked ovary-biased genes and the testis-biased genes described above (Figure 2C), we predicted that if there were even a mild tendency for a faster-X effect on dN/dS in this taxon, it would be readily apparent in this group of genes. However, we found no significant difference in dN/dS values between X-linked and autosomal gonadally unbiased genes (MWU-test *P* > 0.05 Figure 2C; 95% confidence intervals of the medians were 0.0738-0.1163 and 0.0843-0.0874 respectively). Rather, we observed an X/A_dN/dS (gonadally unbiased)_ ratio of 1.08, indicating highly similar dN/dS between these two groups (Figure 2E). In this regard, we conclude that a faster-X effect is not detectable in gonadally unbiased genes.

Finally, we assessed the GT-unbiased genes, and found evidence for greater constraint on the sequence evolution of these genes on the X chromosome as compared to autosomes (X/A_dN/dS (GT-unbiased)_ =0.77, MWU-test *P* < 0.05, Figure 2DF). As expected, however, given that a majority of X-linked genes under study were ovary-biased (Figure 2A, Table S2), and that most genes expressed in the GT-soma were not sex-biased (Figure 2B), many of the X-linked GT-unbiased genes (N = 396) were also ovary-biased (N = 213). Excluding these genes, so that we could consider only those 183 GT-unbiased genes that were not ovary-biased, we found no differences in dN/dS values for these genes between the X chromosome and autosomes (MWU-test *P* > 0.05). In fact, the X/A_dN/dS (GT-unbiased)_ ratio for these GT- and ovary-unbiased genes was 1.05, nearly identical to that observed for gonadally unbiased genes (Figure 2EF). Thus, the GT-somatically unbiased genes, whether they were co-biased in the ovaries or not, exhibited no signals of a faster-X effect.

Taken together, the collective results in Figure 2 show that the absence of a faster-X effect observed here in *Tribolium* largely co-occurs with slow evolution of the abundant X-linked ovary-biased genes, with some contributions from the relatively smaller number of testis-biased, GT-male biased, and GT-female-biased genes (Figure 2C-F). Crucially, the faster-X effect was not even observed in either gonadally-unbiased or GT-soma-unbiased genes, which each yielded an effective X/A_dN/dS_ ratio approaching 1. This latter finding cannot be explained by slow evolution of X-linked sex-biased genes, suggesting that other factors likely also contribute toward the lack of the faster-X effect observed using dN/dS in this taxon (see the below section “*Lack of dosage compensation in the T. castaneum testis*”).

### Why do X-linked ovary-biased genes evolve slowly?

We further considered why the X-linked ovary-biased genes evolved slowly in this taxon (Figure 2C and E). The low dN/dS values observed for ovary-biased genes on the X chromosome (Figure 2C and E) as compared to autosomes suggests that they could be essential genes subjected to high purifying selection, and their ovary-biased expression suggests that they may be involved in female reproduction and thus fitness. To examine this, we determined the predicted GO functions (see Methods: GO functions determined in DAVID (Huang Da *et al.* 2009)) of the ovary-biased genes located on the X chromosome (Figure 2A). Indeed, in agreement with this hypothesis, we found that ovary-biased genes on the X chromosome were enriched for genes involved in ovarian follicle development and *wnt* signaling (Table 2), which is crucial for ovarian development and function in multiple animals (see (Vainio *et al.* 1999; Hernandez Gifford 2015; Naillat *et al.* 2015; Wang *et al.* 2015; Mottier-Pavie *et al.* 2016; Chen *et al.* 2017; Dai *et al.* 2017; Kim-Yip and Nystul 2018; Wang and Page-Mccaw 2018; Bothun and Woods 2019) for examples). X-linked ovary-biased genes also included those with predicted roles in female meiosis and oocyte function (Table 2). These essential ovarian roles were not among the top functional categories observed for ovary-biased genes on autosomes (Table 2). Given these results, we suggest that high purifying selection on ovary-biased genes on the X chromosome is likely at least partly due to the important female reproductive roles of some of these genes.

**Table 2:** Gene ontology (GO) clustering of ovary-biased genes located on the X chromosome and on autosomes. The top clusters with the greatest enrichment scores are shown per category. *P*-values are from a modified Fisher’s test, wherein lower values indicate greater enrichment. Data are from DAVID software (Huang Da *et al.* 2009) using those genes with *D. melanogaster* orthologs

Ovary-Biased Genes on X Chromosome		Ovary-Biased Genes on Autosomes*^a^*	
**Cluster 1: Enrichment Score 3.09**	P-value	**Cluster 1: Enrichment Score 3.56**	**P-value**
Wnt signaling pathway	4.20E-06	Metal-binding	6.00E-05
Segmentation polarity protein	8.20E-05	Zinc ion binding	5.50E-04
Regulation of Wnt signaling pathway	1.60E-04	Zinc-finger	6.60E-04
Segment polarity determination	1.30E-03		
Ovarian follicle cell development	6.70E-03	**Cluster 2: Enrichment Score 2.81**	
Somatic stem cell population maintenance	2.50E-02	Pleckstrin homology-like domain, signaling	1.80E-04
Heart development	3.90E-02	Pleckstrin homology domain, signaling	4.90E-04
**Cluster 2: Enrichment Score 2.92**			
ATP-binding	2.00E-04	**Cluster 3: Enrichment Score: 2.78**	
Nucleotide-binding	3.70E-04	SH2 domain, oncoproteins, signaling	1.40E-04
Nucleotide phosphate-binding region: ATP	1.60E-03	SH3 domain, intracellular or membrane-associated proteins	2.10E-04
Protein kinase, ATP binding site	7.70E-03		

a Data were pooled for all nine autosomes and also includes genes yet unmapped in the genome.

We next considered whether expression breadth could explain the slow evolution of X-linked ovary-biased genes. It is thought that greater expression breadth across tissues, which reflects pleiotropic functionality (Mank and Ellegren 2009), is connected to strong purifying selection and restricts adaptive evolutionary potential, thereby slowing protein evolution (Otto 2004; Zhang *et al.* 2007; Larracuente *et al.* 2008; Mank *et al.* 2008; Mank and Ellegren 2009; Meisel 2011; Assis *et al.* 2012; Harrison *et al.* 2015; Dean and Mank 2016). For example, the slower evolution of female-biased genes than male-biased genes, as reported in various animals (including herein, MWU-test *P* < 0.001 for dN/dS of all ovary-biased *vs.* all testis-biased genes, Figure 2C), may result from their high pleiotropy (Ellegren and Parsch 2007; Assis *et al.* 2012; Parsch and Ellegren 2013; Harrison *et al.* 2015). Indeed, we found here that expression breadth across the four diverse tissue-types (Table S1) was lower for testis-biased than for ovary-biased genes. Specifically, only 25.5% of testis-biased genes (pooled for X-linked and autosomal) were expressed in all four divergent male and female tissue types (at >1FKPM) while 72.8% of ovary-biased genes were transcribed in all four tissues, a pattern concurring with that observed for the male and female gonads in *Drosophila* (Meisel 2011; Assis *et al.* 2012; Whittle and Extavour 2019). In this regard, ovary-biased genes as a group exhibit high pleiotropy, suggesting that their putative roles across multiple tissues may contribute to their slow evolution, via strong purifying constraint and low rates of adaptive evolution (Otto 2004; Larracuente *et al.* 2008; Assis *et al.* 2012; Harrison *et al.* 2015).

It is worth noting that broad expression breadth was observed for ovary-biased genes independently of chromosomal location (78.9% of X-linked ovary-biased genes and 71.6% of autosomal ovary-biased genes were expressed in all tissues). However, the high concentration of ovary-biased genes on the X chromosome as compared to autosomes (Figure 2A) makes pleiotropy a particularly significant factor reducing overall dN/dS for this chromosome (Figure 2C). The slow evolution of X-linked ovary-biased genes is also likely mediated by their involvement in core fitness-related functions (Table 2).

### Lack of dosage compensation in the T. castaneum testis

In X/Y sex determination systems, it has been posited that mechanisms should exist to ensure that the chromosome-wide expression levels of genes on the X chromosome (X) and autosomes (A) would be approximately equivalent in both males (with hemizygous X) and females (homozygous X), such that the ratio of expression of X/A in each sex should equal one (Prince *et al.* 2010; Akoglu 2018). In turn, it may be expected that the chromosome-wide X_male_/X_female_ = A_male_/A_female_ = 1 (Prince *et al.* 2010). Mechanisms for acquiring elevated expression on the single male X chromosome, or dosage compensation, are highly variable and full dosage compensation is sometimes, but not always, achieved (Vicoso and Bachtrog 2009; Mank *et al.* 2010b; Prince *et al.* 2010; Mahajan and Bachtrog 2015; Akoglu 2018). Recently in *D. melanogaster*, it was explicitly shown that dosage compensation in the testis was weak using testis-ovary expression analyses (Argyridou and Parsch 2018). In one prior study of gene expression using microarrays of whole males *vs.* whole females in *T. castaneum* (Prince *et al.* 2010), it was reported that males exhibited full X chromosome dosage compensation, with X_male_/A_male_ = 1.0 and that females exhibited overexpression of the X chromosome, with X_female_/A_female_ = 1.5, thereby yielding X_male_/X_female_ = 0.79 and A_male_/A_female_ =1. Those results were interpreted as evidence that the genes on the X chromosome exhibited complete dosage compensation in males (meaning that expression of the hemizygous X linked genes was equalized to expression of autosomal genes in males), and were overexpressed in females as an imperfect response to dosage compensation (Prince *et al.* 2010). However, a different study that examined published RNA-seq data for somatic glandular tissues in *T. castaneum* did not find evidence for hypertranscription of the X chromosome in females (Mahajan and Bachtrog 2015). Thus, more data are needed on dosage compensation in *T. castaneum*, particularly genes expressed in the gonads, which play key fitness roles, and in the GT-somatic tissues.

**Figure 3:**
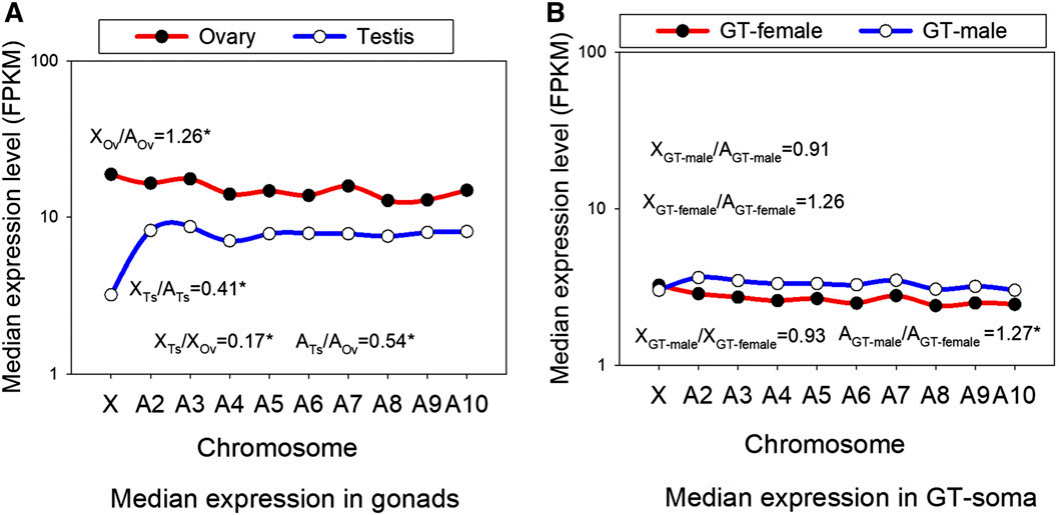
Median expression in the male and female tissues on each of the ten chromosomes in *T. castaneum* for genes with orthologs. A) Gonads; B) GT-soma. For panel A, the ratio of median expression on the X chromosome (X) and autosomes (A) for testis-biased genes and for ovary-biased genes are shown (X_Ts_/A_Ts_ and X_Ov_/A_Ov_). Also shown are X_Ts_/X_Ov_ and A_Ts_/A_Ov_. Panel B contains the equivalent results for the GT-soma. *Indicates a statistically significant difference between the two groups contained in each ratio using MWU-tests.

In Figure 3, we show the median expression level (FPKM) for genes on the X chromosome and each of the nine *T. castaneum* autosomes for the gonads (A) and for the GT-soma (B) using genes that had high-confidence *T. freemani* orthologs (results for all *T. castaneum* genes are in Figure S4, showing similar patterns; note any genes unmapped to chromosomes in the annotation were excluded). We report that expression levels in ovaries (Ov) were largely similar across the nine autosomes (median 14.7 FPKM across nine autosomal medians) and were relatively elevated on the X chromosome (18.8 FPKM, MWU-test *P* = 0.023 of the X chromosome *vs.* autosomes, Figure 3A; note that X/A is measured using multiple decimal places), yielding X_Ov_/A_Ov_ of 1.26 and is consistent with overexpression of X-linked genes in the ovary. For the testis (Ts), however, while expression was also largely similar across all nine autosomes (median 7.9 FPKM across nine autosomal medians), a strikingly lower expression level was observed for the X chromosome (3.2 FPKM, Figure 3A), giving an X_Ts_/A_Ts_ value of 0.41. Thus, there is 2.5 fold lower testis expression of X-linked than autosomal genes (MWU-test *P* < 0.001, Figure 3A; see also Figure S4A where the value was also <0.5), inconsistent with hypertranscription of the single X chromosome in males, at least for the testis-expressed genes. This weak or absent dosage compensation in the *T. castaneum* testis is even beyond recently available comparable data for the testis of *Drosophila*, which had an 0.65 value for this parameter (Argyridou and Parsch 2018). Further, the low value potentially not only suggests a widespread lack of hyperexpression on the X chromosome in the hemizygous state (to balance autosomes) in testis, but could also be consistent with an active mechanism of suppression of X-linked expression during male germ line development (Vibranovski *et al.* 2009; Kemkemer *et al.* 2014; Argyridou and Parsch 2018) in this beetle.

Moreover, we found that testis expression was lower than ovary expression across all nine autosomes, such that A_Ts_/A_Ov_ was equal to 0.54 (MWU-test *P* < 0.001 of autosomal testis to ovary expression, Figure 3A), differing from the equal male/female expression typically expected on autosomes (Prince *et al.* 2010; Argyridou and Parsch 2018). This effect was even more pronounced for the X chromosome, where X_Ts_/X_Ov_ had a value of 0.17 (Figure 3A, MWU-test *P* < 0.001 for X chromosome testis expression *vs.* ovary expression), indicating that even after taking into account the lower expression level observed on all autosomes for testis genes *vs.* ovary genes (median 1.9 fold), testis genes exhibited a greater drop (5.9 fold) in expression on the X chromosome. In this regard, both X_Ts_/A_Ts_ and X_Ts_/X_ov_ (Figure 3A) suggest a significant lack of testis dosage compensation in this beetle.

For the GT-soma, we observed nearly perfect dosage compensation on the X chromosome for GT-males, both with respect to GT-female expression levels, such that X_GT-male_/X_GT-female_ =0.93 (median of 3.02 and 3.25 FPKM respectively MWU-test *P* = 0.74), and with respect to autosomal GT-male expression levels, with X_GT-male_/A_GT-male_ = 0.91 (MWU-test *P* = 0.11). Thus, unlike genes expressed in the testis, genes expressed in the non-gonadal tissues of males (GT-males) exhibited substantial dosage compensation (Figure 3B, Figure S4B). The median GT-male expression across all nine autosomes was consistently higher than the median expression in GT-females, yielding A_GT-male_/A_GT-female_ of 1.27 (MWU-test *P* < 0.001), a trend opposite to the higher expression level observed for ovary genes relative to testis genes (Figure 3AB). Nonetheless, GT-female genes on the X chromosome were expressed at substantially higher levels than such genes on autosomes, yielding X_GT-female_/A_GT-female_ = 1.26 (MWU-test *P* = 0.064, note above 0.05), and thus contributing to the observed highly similar expression levels between GT-females and GT-males on the X chromosome. In sum, the GT-males show evidence of nearly complete dosage compensation, differing markedly from the testis. Additional study of more individual somatic tissues (*e.g.*, brain, hindgut), similar to that in other recent studies (Mahajan and Bachtrog 2015; Argyridou and Parsch 2018), will be needed to assess whether the variation in GT-female expression among autosomes is observed in various somatic tissue types in *T. castaneum*.

### Lack of testis dosage compensation and sex-biased gene expression

The striking tendency for weak or absent dosage compensation in the testis, and a relatively modest overexpression in the ovary (Figure 3A), are consistent with the high concentration of ovary-biased genes that were observed on the X chromosome (Figure 2A, Figure S3A) in this taxon. While ovary-biased genes (≥twofold sex-bias in expression) on the X chromosome by definition lack equal male-female expression, or dosage compensation, and the rare testis-biased genes exhibit overexpression on the X chromosome in males, the gonadally unbiased genes should be expected to have the greatest similarity in FPKM between males and females (<twofold difference, and/or *P* > 0.05). However, we found that that only 11.5% of the 190 unbiased genes located on the X chromosome (Table S2) had any level of higher expression in males than females (88.5%, sign test *P* < 10^−5^), suggesting that testis X chromosome dosage compensation is largely precluded even in the unbiased genes. Collectively, given that the faster-X effect may be anticipated to be strongest in taxon groups with complete dosage compensation, due to elevated phenotypic protein product and effects of beneficial recessive mutations in males (Charlesworth *et al.* 1987; Mank *et al.* 2010b; Pinharanda *et al.* 2019), the overall lack of testis dosage compensation in *T. castaneum* (Figure 3A) suggests it may significantly contribute to the absence of the faster-X effect observed in this taxon (Figure 1, Figure 2) by minimizing the phenotypic effects of recessive beneficial mutations.

## Discussion

### Absence of a faster-X effect based on dN/dS in beetles

Our results show the absence of a faster-X effect on protein sequence evolution, and a tendency for slower evolution on the X chromosome than autosomes, in this *Tribolium* system (Figure 1). Thus, this result differs from those reported for most other organisms studied to date and suggests that the two main possible causes of a faster-X effect, namely relaxed selection on the X chromosome and/or the rapid fixation of beneficial mutations in hemizygous males (Charlesworth *et al.* 1987; Mank *et al.* 2010a; Meisel and Connallon 2013; Parsch and Ellegren 2013; Campos *et al.* 2018), are not primary factors shaping evolutionary rates on the X chromosome in this taxon. The former mechanism is excluded given that an absence of a faster-X effect in these beetles in itself demonstrates that unlike certain organisms such as birds (Mank *et al.* 2010a; Mank *et al.* 2010b; Parsch and Ellegren 2013), relaxed selection (due to effective population size effects) does not broadly accelerate evolution of protein coding genes on the X chromosome as compared to autosomes. Moreover, our data are also suggestive of a limited history of positive selection on the X chromosome given that: 1) we did not observe an elevated faster-X effect in testis- or GT-male- biased genes as compared to their female-counterparts or unbiased genes, as would be expected under fixation of recessive beneficial mutations (Baines *et al.* 2008; Mank *et al.* 2010a; Parsch and Ellegren 2013), and rather a faster-X effect was not observed in any studied group (Figure 2C-F); 2) the abundant and slowly evolving X-linked ovary biased genes exhibited high pleiotropy (78.9% were expressed in all four diverse tissues, and had core female fitness-related functions, Table 2), a factor thought to increase the strength of purifying selection (as we observed, Figure 2C), and to largely restrict adaptive evolution (Otto 2004; Zhang *et al.* 2007; Larracuente *et al.* 2008; Mank *et al.* 2008; Mank and Ellegren 2009; Meisel 2011; Assis *et al.* 2012; Harrison *et al.* 2015; Dean and Mank 2016) and; 3) there was a striking lack of testis dosage compensation in this taxon, a factor thought to impede the fixation of recessive beneficial mutations (Charlesworth *et al.* 1987; Mank *et al.* 2010b). Collectively, it is evident that our results appear to exclude an extensive history of either positive selection (Charlesworth *et al.* 1987; Meisel and Connallon 2013) or relaxed selective constraint (Mank *et al.* 2010a; Parsch and Ellegren 2013) acting on the X chromosome at a level that is sufficient to cause a faster-X effect detectable at the protein sequence level (dN/dS) in this beetle taxon.

*Tribolium* remains an understudied evolutionary system as compared to the predominant insect genomics model *Drosophila* (Meisel and Connallon 2013; Campos *et al.* 2018; Charlesworth *et al.* 2018), in which the availability of multiple population level genomic datasets and multispecies genomes has allowed intensive study of the faster-X effect using a wider range of approaches than are currently available for *Tribolium* (Baines *et al.* 2008; Kayserili *et al.* 2012; Meisel and Connallon 2013; Avila *et al.* 2014; Charlesworth *et al.* 2018). Thus, attaining additional population data and whole genomes of multiple *Tribolium* species will be valuable for allowing further tests of putative positive selection on the X chromosome using complementary approaches to the ones employed here. These methods could include, for example, McDonald-Kreitman tests (Mcdonald and Kreitman 1991; Meisel and Connallon 2013; Rousselle *et al.* 2016; Murga-Moreno *et al.* 2019; Pinharanda *et al.* 2019) and branch-site analyses of dN/dS in multiple species (Yang 2007; Kosakovsky Pond *et al.* 2019). Population data analyses will also allow measures of recombination rates (Stumpf and Mcvean 2003; Comeron *et al.* 2012), which has been suggested to influence the faster-X effect (Campos *et al.* 2018) and assessments of potential roles of effective population size (Mank *et al.* 2010b). Such studies will help further decipher the mechanisms underlying the lack of a faster-X effect on dN/dS in these beetles.

### Absence of a faster-X effect male-biased and unbiased genes

While it is evident that the abundant X-linked ovary-biased genes evolve slowly and thus are linked to the absent faster-X effect in this taxon (Figure 2C), the relatively rare X-linked testis-biased and GT-male biased genes (Table S2) also showed no consistent tendency toward a faster-X effect (Figure 2CD, Table 1). In these genes, mildly deleterious recessive mutations may be immediately exposed to purifying selection in the hemizygous state in males (Charlesworth *et al.* 1987). Thus, it is possible that a much different mechanism could underlie an absent faster-X effect in those genes, than that operating on ovary-biased genes. For example, the rapid purging of deleterious mutations in males has been suggested to counteract the rapid fixation of X-linked beneficial mutations, and to cause the weak faster-X effect observed in *Drosophila* (Mank *et al.* 2010b). A similar purging mechanism has been suggested to cause the unexpectedly slow evolution of Z-linked female-biased genes in certain butterflies (Rousselle *et al.* 2016). In this regard, purging of deleterious mutations may counter a faster-X effect in testis-biased and GT-male biased genes in *Tribolium*.

With respect to the X-linked unbiased genes, like ovary-biased genes, these genes were very common on the X chromosome (Table S2) and thus were statistically rigorous in showing an absence of a faster-X effect (Figure 2CD, Table 1). The unbiased genes, which are expressed relatively similarly (<2 fold sex-bias) in both sexes may also exhibit an absent faster-X effect due to rapid purging of deleterious mutations in hemizygous males (Charlesworth *et al.* 1987). However, our results suggest that the marked lack of testis dosage compensation even for unbiased genes (88.5% of gonadally unbiased genes had higher expression in ovaries), may reduce the male phenotypes and selection coefficients of recessive beneficial mutations, thus limiting their fixation, and may also act to impede the faster-X effect in these unbiased genes (Charlesworth *et al.* 1987; Mank *et al.* 2010b). Further, this type of mechanism may also partly contribute to the slow evolution of X-linked ovary-biased genes (as most have some degree of expression in testis).

Recent reports from *Drosophila* have shown that dosage compensation is weak or absent for the testis (Argyridou and Parsch 2018), and that this insect exhibits active suppression of X-linked expression in males (Kemkemer *et al.* 2014; Argyridou and Parsch 2018). Consistently, it was found that the transfer of X-linked testis-expressed genes to the autosomes resulted in marked upregulation in *D. melanogaster* (Kemkemer *et al.* 2014), suggesting an active mechanism of suppression of expression on the X chromosome in testis. While the mechanism for X-linked active suppression of expression is unknown, it could reflect male meiotic sex chromosome inactivation (MSCI). Empirical support for MSCI has been observed for *D. melanogaster* (Vibranovski *et al.* 2009; Vibranovski *et al.* 2012), and a strong effect has been found in range of other animal systems including mammals (Turner 2007) and *Caenorhabditis elegans* (Bean *et al.* 2004). Further study will be needed to ascertain whether the absence of dosage compensation in the testes for *T. castaneum* involves lack of upregulation on the X chromosome and/or also includes an active process involving X chromosome suppression or silencing.

Finally, while we propose that the absence of the faster-X effect herein is linked to slow evolution of the abundant X-linked ovary-biased genes and lack of dosage compensation in the testis, we do not exclude a role of standing genetic variation. For instance, large populations tend to contain more polymorphic loci, which can accelerate autosome evolution if adaptation occurs via standing genetic variation rather than *de novo* mutations (Orr and Betancourt 2001; Meisel and Connallon 2013; Charlesworth *et al.* 2018). This phenomenon could possibly occur in beetles, and thus we do not exclude this factor in partly contributing toward the absence of a faster-X effect in this taxon.

### Conclusions and future directions

We have demonstrated the absence of the faster-X effect at the protein sequence level in a *Tribolium* system, that may be explained by at least two putative underlying mechanisms, namely slow evolution of abundant X-linked ovary-biased genes and a lack of testis dosage compensation, limiting fixation of recessive beneficial mutations. Future studies should aim to further test positive and negative selection using the genomes from multiple *Tribolium* species (Yang 2007) and population data from *T. castaneum* (Rousselle *et al.* 2016; Pinharanda *et al.* 2019). A further understanding of dosage compensation may be achieved by attainment of transcriptional data from a wide range of individual somatic tissue types in *T. castaneum*, similar to analyses recently conducted in *Drosophila* (Argyridou and Parsch 2018). Such multi-tissue expression data will also allow further assessments of cross-tissue pleiotropy of sex-biased genes (Mank and Ellegren 2009; Meisel 2011; Whittle and Extavour 2019) and may help further disentangle its role in constraining the evolution of ovary-biased genes (Figure 2).

Moreover, experimental research of MSCI in *T. castaneum*, as has been conducted in other organisms (Reinke *et al.* 2004; Turner 2007; Vibranovski *et al.* 2012), will help reveal whether the lack of dosage compensation observed in the testis is due to transcriptional silencing in the male meiotic cells. In addition, studies using X-linked genes inserted into the autosomes, and vice-versa (Bean *et al.* 2004; Kemkemer *et al.* 2014; Argyridou and Parsch 2018) may help discern the dynamics of dosage compensation in *T. castaneum*. Finally, research on the faster-X effect, including analyses of sex-biased genes and dosage compensation, should be extended to include more understudied organisms, to help reveal the breadth of this phenomenon in metazoans and its underlying mechanisms.
